# Evaluating student use, access, and perceptions of a campus sexual health vending machine

**DOI:** 10.3389/fpubh.2026.1916539

**Published:** 2026-09-10

**Authors:** Grace Sheng, Lisa Gualtieri

**Affiliations:** 1 Tufts University School of Medicine, Boston, MA, United States; 2 Cedars-Sinai Medical Center, Los Angeles, CA, United States

**Keywords:** access to care, contraception, reproductive health, sexual health, university health

## Abstract

**Background:**

Sexual health vending machines have emerged as a promising approach to expanding access to contraception and other sexual health products on college campuses. This study evaluated patterns of use, perceived accessibility, and student attitudes towards the sexual health vending machine at Tufts University in Medford, Massachusetts, United States.

**Methods:**

This mixed-methods evaluation combined an online survey with open and closed-ended responses and vending machine restocking records to contextualize overall utilization. Enrolled students ≥18 years were recruited using flyers and campus social media. Analyses included descriptive analysis, Fisher’s exact tests, and thematic analysis of open-ended responses.

**Results:**

The survey was open from October to December 2025 and included 118 respondents. Self-reported use suggests students engage with the vending machine as needed. Users were significantly more likely than non-users to agree that it increased their likelihood of condom use. However, both groups expressed strong support for the vending machine, citing improved access to sexual health resources, satisfactory product variety, and endorsement of university provision. Although many students appreciated its visibility and role in reducing stigma, privacy and discretion concerns were the most commonly reported reasons for non-use. Restocking records indicated sustained utilization during the academic year.

**Conclusion:**

These findings suggest that the Tufts University sexual health vending machine is a valuable initiative that may improve access to sexual health resources and contribute to the normalization of preventative sexual health behaviors. Its unique combination of immediacy, affordability, and convenience may further enhance its value as a low-barrier resource for students.

## 1 Background

Sexually transmitted infections and unintended pregnancy continue to be public health priorities among adolescents and young adults, with national data underscoring persistent risk behaviors such as low or inconsistent contraceptive use (1). Given the potential lasting physical and psychosocial implications of STIs and unintended pregnancy, understanding patterns of contraception use in this population is an important area of public health research.

College students continue to experience barriers to obtaining timely sexual and reproductive health products. Students frequently reported obstacles including cost, privacy concerns, inconvenient pharmacy or health center hours, embarrassment or stigma related to obtaining sexual health products, and lack of awareness of campus resources (2–4). Such barriers may delay or prevent necessary contraceptive use, particularly in time-sensitive situations requiring emergency contraception. Ensuring rapid, convenient access to sexual and reproductive health resources is a key component of effective pregnancy and STI prevention efforts on college campuses (5).

In response, universities across the United States have begun placing vending machines on campus that dispense contraception and other sexual health products. Reports from 2023 showed that approximately 39 universities in 17 states operated such vending machines (6), a number that has grown to more than 150 campuses as of May 2026 (7).

Tufts University, a private university in Medford, Massachusetts with 7,126 undergraduate and 6,473 graduate students (8), participates in this trend of increasing sexual health access in universities. The first sexual health vending machine was installed in October 2022 by the Center for Awareness, Resources, and Education (CARE) in collaboration with the Tufts student group Sex Health Reps, with the goal of encouraging safe sexual practices and reducing stigma surrounding sexual health (9). The issue of accessibility, only exacerbated by the COVID-19 pandemic, has long been a central concern, so this appointment-free, grab-and-go resource was designed to provide students with a convenient and reliable way to access sexual health products. The vending machine is located on the ground floor of the campus center, a high-traffic student hub. Although positioned in a secluded corner to enhance privacy, it remains readily visible and accessible to students. External and internal condoms, dental dams, and lubricant are available free of charge and can be obtained without a student ID to ensure discretion (10).

Despite the growing implementation of sexual health vending machines on college campuses, published evaluations remain limited. However, the few studies conducted to date have reported high student acceptability and perceived improvements in access to sexual and reproductive health products. Students consistently identify convenience, affordability, and privacy as important advantages (11, 12). Product distribution has also generally aligned with student preferences (11), supporting the feasibility of this intervention as a strategy to expand access to sexual health resources on campus. However, important questions remain regarding patterns of use, barriers to utilization, and how factors such as student population, campus culture, machine location, and pricing influence use.

## 2 Objective

Although these early findings are promising, evidence remains limited and the impact of the Tufts University sexual health vending machine had not been formally assessed. In addition, few studies have combined objective utilization data with student-reported experiences. Therefore, this study evaluated student use of and attitudes toward the Tufts University sexual health vending machine by integrating restocking records with self-reported and other qualitative survey data to provide a more comprehensive understanding of its use, accessibility, and perceived impact.

## 3 Methods

This mixed-methods study at Tufts University deployed an online survey and collected vending machine inventory restocking records. Findings from these data sources were integrated during interpretation to provide a comprehensive evaluation of the vending machine.

An 18-question online survey was developed by the research team in Qualtrics, detailed in the supplementary materials. Closed-ended questions were used to characterize patterns of use and accessibility, while open-ended questions allowed participants to share nuanced experiences, perceptions, and recommendations regarding the vending machine. Demographic data collected included year in school, gender identity, sexual identity/orientation, race/ethnicity, and place of residence during the academic year. The survey took approximately 5 min to complete. All questions required some response to complete and submit the survey, but participants were free to skip questions they felt uncomfortable answering by selecting “N/A.” Prior to implementation, the survey questions were piloted with 3 current Tufts University School of Medicine students who previously attended Tufts University as undergraduates to assess question clarity, relevance, survey flow, and functionality. Based on participant feedback, the questionnaire was streamlined by reducing the number of pages and prioritizing closed-ended response formats to minimize respondent burden and optimize survey completion. The Tufts Social, Behavioral, and Educational Research Institutional Review Board determined that IRB review and approval was not required.

The survey was open from October 30, 2025 to December 12, 2025. Participants were recruited with printed flyers containing a QR code to the survey, which were placed inside vending machine product envelopes. Advertisements were also posted on campus organization social media accounts, including Tufts Sex Health Reps, Planned Parenthood Action at Tufts, and the Tufts Side chat forum. Any student 18 years or older and currently enrolled in classes at Tufts University was eligible to respond. Qualtrics settings were configured to limit responses to one submission per browser/device, reducing the likelihood of duplicate responses. Survey responses contained no identifiable information and demographic characteristics were collected for the purpose of conducting statistical analyses. Upon survey completion, participants were directed to a separate webpage where they could optionally provide their email address to enter a random drawing for a $50 gift card provided by CARE. In this way, email addresses were collected independently from survey responses to maintain anonymity.

Survey data was analyzed using StataSE. Closed-ended questions were analyzed using descriptive statistics, including count and frequencies. Demographic data were analyzed using Fisher’s exact tests to assess for statistically significant differences between subgroups. Statistical significance was defined as a *p* value less than or equal to 0.05. When appropriate, Likert-scale response categories were collapsed to reduce sparse cells and facilitate Fisher’s exact testing. For instance, “strongly agree” and “somewhat agree” were combined as “agree,” and “strongly disagree” and “somewhat disagree” were combined as “disagree,” with “neither agree nor disagree” retained as a separate category. We also conducted thematic analysis of open-ended questions to identify recurring patterns regarding student experiences and attitudes. N/A responses were excluded from analyses on a question-by-question basis; thus, the number of valid responses for each item varied.

Machine restocking records were collected from January 14, 2025 to December 5, 2025. Counts of products restocked were provided by CARE and contained no identifying information.

## 4 Results

### 4.1 Demographics

A total of 118 students completed the survey. Participants were primarily undergraduate students (91.5%), composed of 16.4% freshmen, 38.8% sophomores, 22.4% juniors, and 15.5% seniors. The sample was predominantly female (77.4%) and included a diversity of sexual identities, with just over half identifying as heterosexual (53.0%). Approximately half of respondents identified as White (51.8%), followed by Asian (22.8%), Hispanic/Latino (12.3%), and multiracial (8.8%). Most participants resided on campus (62.1%) or off campus in the surrounding area (36.2%). These data are presented in detail in Table 1.

**Table 1:** Participant demographic characteristics.

Characteristic	Total
Year in school (*N* = 116)	
Freshman (1st year)	19 (16.4%)
Sophomore (2nd year)	45 (38.8%)
Junior (3rd year)	26 (22.4%)
Senior (4th year)	18 (15.5%)
Graduate student	7 (6.0%)
Other	1 (0.9%)
Gender identity (*N* = 115)	
Male	21 (18.3%)
Female	89 (77.4%)
Non-binary	5 (4.4%)
Sexual identity/orientation (*N* = 115)	
Heterosexual	61 (53.0%)
Bisexual	30 (26.1%)
Gay/Lesbian	11 (9.6%)
Pansexual	6 (5.2%)
Asexual	1 (0.9%)
Other	6 (5.2%)
Race/ethnicity (*N* = 114)	
White	59 (51.8%)
Asian	26 (22.8%)
Hispanic/Latino	14 (12.3%)
Black/African American	2 (1.8%)
Native Hawaiian/Pacific Islander	1 (0.9%)
Middle Eastern/North African	2 (1.8%)
Multiracial/Mixed	10 (8.8%)
Place of residence (*N* = 116)	
On campus dorm/apartment	72 (62.1%)
Off campus—Surrounding area	42 (36.2%)
Off campus—Commuter	2 (1.7%)

Demographic characteristics of participants who completed the sexual health vending machine survey, including academic status, gender identity, sexual orientation, race/ethnicity, and residence status (*N* = 114–116, depending on item response).

### 4.2 Vending machine use

Of the 118 respondents, almost all (98.3%) were aware of the vending machine prior to taking the survey. Many of the respondents learned about the vending machine through campus promotion (61.4%), while others heard about it through friends (24.6%), social media (21.1%), seeing it in the campus center (19.3%), or through resident assistants (4.4%).

Most participants (66.7%) reported not having used the vending machine in the past 30 days. An additional 23.1% reported using it once, while 10.2% used it two times or more. Among vending machine users, 70.0% obtained condoms (external or internal), 40.0% obtained lubricant, 7.5% obtained dental dams, and 40.0% obtained other products. When asked about the circumstances for obtaining vending machine products, students most commonly reported “to keep on hand just in case” (*n* = 40), 25 “in response to an unplanned need,” and 20 “right before sexual activity.” Vending machine use did not differ significantly between those that lived on and off campus (*p* = 0.268).

### 4.3 Accessibility and barriers

When asked if they have ever wanted to use the machine but decided not to, 38.1% of respondents answered “yes.” Of those, the most cited reason was visibility/privacy concerns (81.8%), followed by not knowing how to use the machine (10.9%), cost (9.1%), and the desired product being out of stock (1.8%).

Most respondents reported they would obtain sexual health products from a pharmacy (83.2%), campus health center (31.9%), or resident assistant (17.7%) if the vending machine was not available on campus. Other answers included online (12.4%), from friends (7.1%), or other (1.8%).

Over half of respondents felt that having the vending machine on campus increased their likelihood of using a condom, with 32.4% strongly agreeing and 36.3% somewhat agreeing. 26.5% neither agreed nor disagreed, 1.0% somewhat disagreed, and 3.9% strongly disagreed Furthermore, students who reported using the vending machine in the past 30 days were more likely to endorse this effect than non-users (RR = 1.41, 95% CI: 1.10–1.81; *p* = 0.014).

### 4.4 Perceptions and attitudes

There was a very strong consensus among respondents that the vending machine makes it easier to obtain sexual health products, with 77.6% strongly agreeing, 20.7% somewhat agreeing, only 1.7% neither agreeing nor disagreeing, and no respondents disagreeing. This did not differ significantly by vending machine use (*p* = 0.548), with 100% of users and 97.4% of non-users endorsing the statement.

Most participants were either very satisfied (64.4%) or somewhat satisfied (25.0%) with the variety of products offered in the vending machine, with only 9.6% neither satisfied nor dissatisfied and 1.0% somewhat dissatisfied. This did not differ by vending machine use (*p* = 0.227), with 92.3% of users and 87.7% of non-users reporting feeling satisfied. This also did not differ by year in school (*p* = 0.398), gender identity (*p* = 0.685), sexual identity/orientation (*p* = 0.594), race/ethnicity (*p* = 0.792), or place of residence (*p* = 0.648).

Nearly all respondents felt Tufts should be providing sexual health resources in this way, with 88.1% indicating they felt it was very important and 11.0% somewhat important. Only 1 respondent selected ‘N/A’ and no respondents rated the approach as unimportant; therefore, inferential comparisons by vending machine use were not conducted.

The main comparative analyses are summarized in Supplementary Tables S1, S2.

### 4.5 Participant feedback and suggestions

In response to an open-ended question about additional products, participants most commonly suggested different condom sizes (*n* = 6), greater lubricant variety (*n* = 4), and menstrual products (*n* = 4); less frequently mentioned items included spermicide, diaphragms, sex toy wash, and drink-sticker lids (each *n* = 1). Regarding another open-ended question about experiences with the vending machine, participants most frequently commented on discretion and cost. Some expressed appreciation for reduced cost (*n* = 8), convenience (*n* = 4), and discretion (*n* = 4), while others raised concerns about insufficient discretion (*n* = 11); a small number described the machine as either easy or confusing to use (each *n* = 1). Several respondents also described the vending machine as contributing to reduced stigma and increased visibility of sexual health resources on campus, noting that “having it as an option and seeing others use it has made it less stigmatized and accessible for people” and that they appreciated it as “a resource and visible marker of sex positivity/education on campus.”

### 4.6 Vending machine restock data

**Figure 1:**
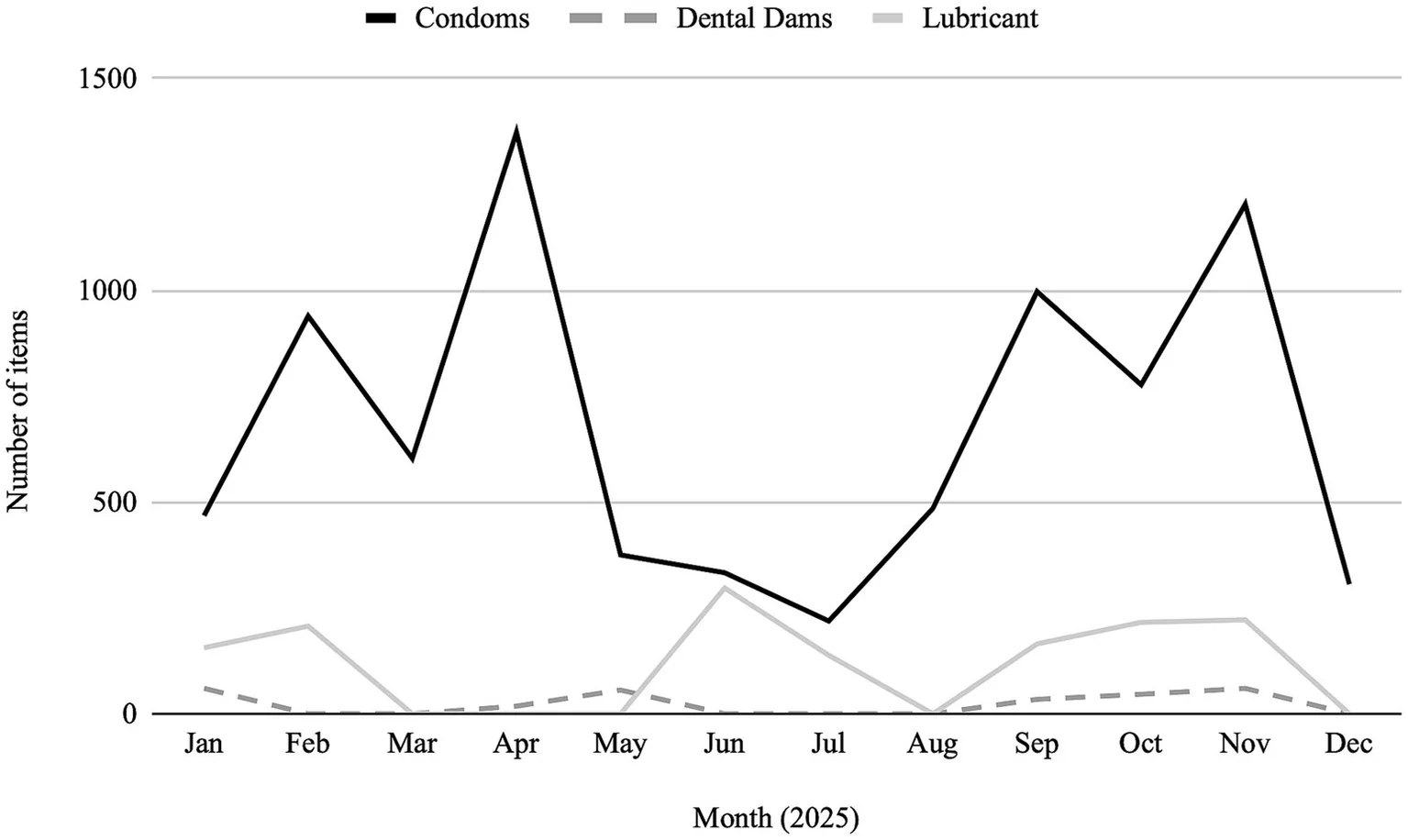
Monthly restocking of sexual health vending machine products. Monthly counts of restocked condoms (external and internal), dental dams, and lubricant in the campus sexual health vending machine over a 12-month period.

Monthly restock volumes varied but indicated consistent utilization of the vending machine across the calendar year, with expected decreases corresponding to when campus is less populated (summer and winter academic breaks). Condoms were restocked in greater quantity and more frequently than lubricant or dental dams (Figure 1).

## 5 Discussion

In this study, the majority of students reported not having used the sexual health vending machine in the past 30 days. However, perceptions of the resource were overwhelmingly positive and outweighed the proportion of recent vending machine users. Over 90% of participants agreed that the vending machine made sexual health products easier to obtain, were satisfied with the variety of products offered, and felt that the university should provide sexual health resources in this manner. This distinction suggests that the perceived value of the vending machine extends beyond direct utilization, with recognition of its role as a campus sexual health resource benefiting the broader student body.

Although most respondents reported no use of the vending machine in the past 30 days, this pattern likely reflects the episodic nature of sexual health products rather than lack of utility. Longitudinal research among young adults has demonstrated that sexual activity varies over time and that contraceptive use changes in relation to sexual activity (13). Consistent with this, students in our sample most commonly reported obtaining products to keep on hand “just in case,” in response to an unplanned need, or immediately before sexual activity. Restocking records demonstrated very consistent replenishment throughout the year, apart from expected declines during vacation periods, supporting the role of the vending machine as a reliable resource when needed. Moreover, restocking data from the survey period (October 30 to December 12, 2025) substantially exceeded self-reported use captured by the survey—with 1,509 condoms, 60 dental dams, and 222 units of lubricant replenished in November and December—suggesting that individual-level survey responses largely underestimate overall vending machine utilization.

Most participants reported being very satisfied or somewhat satisfied with the variety of products offered in the vending machine, with no significant differences between recent users and non-users or among any demographic characteristic. These findings indicate that the current product selection is adequately serving a diverse range of students, consistent with prior evaluations of other vending machines (11, 14). Participant suggestions for additional products—including different condom sizes, expanded lubricant options, and menstrual products—point to opportunities for further aligning the vending machine with student needs and enhancing the inclusivity of the resource.

Over half of respondents indicated that having the vending machine on campus increased their likelihood of using a condom, with recent users significantly more likely to endorse this effect than non-users. This finding is consistent with other condom distribution studies demonstrating that access to low-barrier, readily available resources may support safer sexual behaviors among those who engage with them (15, 16). Notably, the proportion of respondents endorsing this effect exceeded the number of students who had used the vending machine in the past 30 days, suggesting that the machine’s impact may extend beyond direct use. This finding raises the possibility of a broader ripple effect described in other studies, in which the presence of visible sexual health resources may shape attitudes and intentions related to contraception use at a larger population level, even among non-users (12, 17, 18).

Together with high levels of perceived utility and satisfaction, students’ strong agreement that the vending machine improves access to sexual health products further supports its potential to serve as both an access point and visible signal that sexual health is a routine aspect of overall health and well-being. Participants described the machine as reducing stigma, normalizing sexual health resource use, and contributing to an overall more supportive campus environment, consistent with other vending machine and sexual health service evaluations (12, 18). However, this same visibility was identified as a barrier to use, with some respondents expressing concern about being seen obtaining products. This tension between promoting accessible sexual health resources and preserving individual privacy was also highlighted in Sanyaolu et al. (12).

Most respondents indicated that they would obtain sexual health products from a local pharmacy if the vending machine was unavailable. Despite the availability of online purchasing, relatively few chose this as a preferred alternative, suggesting discretion alone may not fully explain use patterns. Instead, the vending machine appears to offer a unique combination of immediacy, affordability, and convenience that is not fully replicated by existing alternatives, consistent with other vending machine evaluations (11, 12). These benefits include extended operating hours, elimination of the need for appointments, and free or lower-cost access to essential products, which may be particularly important for students with limited flexibility to access healthcare services during standard business hours.

This is further supported by the finding that vending machine use did not differ by residence. Although Houlihan et al. (11) found that students living on campus were more likely to perceive the intervention as improving access to sexual health products, our findings suggest that this may not translate into differences in actual utilization. Even among off-campus students, who may have greater access to pharmacies and other retail sources, vending machine use remained comparable to that of the on-campus group, suggesting that the resource provides benefits beyond geographic convenience alone. The vending machine may also provide an alternative for students who face discomfort, stigma, or privacy concerns in traditional clinical settings, which have been identified as barriers to accessing sexual and reproductive health services among young adults (19, 20).

This study has several limitations. Survey responses were self-reported and may be subject to recall or social desirability bias. Although Qualtrics settings were used to limit responses to one submission per browser/device, duplicate responses from individuals using multiple devices or browsers could not be completely excluded. Additionally, the cross-sectional survey design and potential for unmeasured confounding variables limit causal conclusions regarding the vending machine’s effects on access or sexual health behaviors. While the sample reflects a diverse student body, recruitment relied on convenience sampling at a single institution and may not be representative of the broader US college student population. As such, the generalizability of these findings to institutions with different demographic profiles, campus cultures, or resource availability may be limited. Additionally, vending machine restock data reflect aggregate replenishment rather than individual use patterns and cannot be directly linked to survey respondents. The vending machine represents only one potential source of sexual health products, so utilization should not be interpreted as a direct measure of students’ overall access to or use of sexual health products.

Despite these limitations, this study contributes to a growing body of evidence that sexual health vending machines may represent a promising strategy for increasing access to sexual health products while fostering awareness and reducing stigma surrounding their use. Future research should incorporate longitudinal and more rigorous qualitative methods, such as interviews or focus groups, to evaluate vending machines across diverse institutional settings to better understand the generalizability of these findings.

## 6 Conclusion

This study provides evidence on the utilization and perceived value of the Tufts University campus sexual health vending machine. Patterns of self-reported use suggest that individuals engage with the resource on an as-needed basis, consistent with the inherently situational nature of sexual health needs. However, restocking records demonstrate steady and substantial utilization throughout the study period and over time. Together, these results indicate that the vending machine serves as a valuable and accessible complement to existing campus health services and may contribute to the normalization of sexual health resource use. Given that visibility functioned as both a facilitator and a barrier to use, future initiatives could consider expanding access through additional locations across the Tufts campus, including more private settings that offer greater discretion while maintaining the visibility and awareness benefits of a centrally located machine. Overall, these findings suggest that the Tufts University sexual health vending machine may offer a promising approach to reducing barriers to preventive care, expanding access to sexual health resources, and supporting health-promoting norms among college students. Further research across diverse institutions will be important to determine whether these benefits can be replicated and sustained on a broader scale.

## Data Availability

The datasets presented in this article are not readily available due to privacy and sensitivity concerns. Requests to access the datasets should be directed to grace.sheng@tufts.edu.
