# Supplementary material for: Evaluating student use, access, and perceptions of a campus sexual health vending machine
Source: Front Public Health. 2026 Sep 10;14:1916539. doi: 10.3389/fpubh.2026.1916539 (PMC13601293; doi:10.3389/fpubh.2026.1916539)
Supplement: Supplementary file 1 [file Data_Sheet_1.docx]

Researchers at Tufts University School of Medicine in Massachusetts are asking you to participate in research on the sexual health vending machine on the Tufts Medford campus. We will be asking you a set of questions to gain a better understanding of your awareness and use of this resource.

Participation is voluntary. All questions must have some response in order to complete and submit the survey, but you can skip any questions that you do not feel comfortable answering by selecting or answering "N/A". At the end of the survey, there will be an option for you to provide your email address if you would like to be entered into a drawing to receive a $50 Amazon gift card. If more than 100 people enter, your odds of winning are less than 1 in 100. Winning is not guaranteed.

To maintain your privacy, no identifying information will be disclosed, and all records will be stored on secure servers. If you choose to provide your email address to be entered into the raffle, your email address will be deleted once all raffle winners are notified. Although it is possible that your data could be seen by someone outside of the study team, we do not anticipate this or other risks to you. There are no benefits to you for participating.

If you have any questions, please contact the study PI, Grace Sheng at grace.sheng@tufts.edu. If you have questions or concerns about your rights as a research participant, or if you would like to discuss the study with someone outside of the research team, contact Tufts University Social Behavioral & Educational Research Institutional Review Board via telephone (617-627-8804) or email (sber@tufts.edu). You must be a student currently enrolled at Tufts University and 18 years old or older to participate in this study.

**Part 1: Use**

1. How many times have you used the vending machine in the past 30 days?
   a. 0 times
   b. 1 time
   c. 2-3 times
   d. 4-5 times
   e. 6 or more times
   f. N/A
2. If applicable, which items did you obtain? (Select all that apply)
   a. Internal condom
   b. External condom
   c. Dental dam
   d. Lubricant
   e. Other
   f. N/A
3. Do you usually get products from the vending machine… (Select all that apply)
   a. To keep on hand “just in case”
   b. Right before sexual activity
   c. In response to an unplanned need
   d. Other: [Open text]
   e. N/A
4. Please indicate how much you agree or disagree with the following statement:
   Having the vending machine on campus has increased my likelihood of using a condom.
   a. Strongly agree
   b. Somewhat agree
   c. Neither agree nor disagree
   d. Somewhat disagree
   e. Strongly disagree
   f. N/A

**Part 2: Accessibility and Barriers**

5. Have you ever wanted to use the machine but decided not to?
   a. Yes
   b. No
   c. N/A
6. If yes, what were the reasons? (Select all that apply)
   a. Cost
   b. Visibility/privacy concerns
   c. Machine out of stock
   d. Didn’t know how to use it
   e. Other: [Open text]
   f. N/A
7. If there wasn’t a vending machine on campus, where would you most likely obtain sexual health products? (Select all that apply)
   a. Campus health center
   b. Pharmacy (e.g. CVS, Walgreens)
   c. Online
   d. Resident assistant (RA)
   e. Friends
   f. Other: [Open text]
   g. N/A
8. How did you learn about the vending machine? (Select all that apply)
   a. Campus promotion
   b. Social media
   c. Friends
   d. Resident assistant (RA)
   e. I wasn’t aware before today
   f. Other: [Open text]
   g. N/A

**Part 3: Perceptions and Attitudes**

9. Please indicate how much you agree or disagree with the following statement:
   The vending machine makes it easier to get sexual health products.
   a. Strongly agree
   b. Somewhat agree
   c. Neither agree or disagree
   d. Somewhat disagree
   e. Strongly disagree
   f. N/A
10. How satisfied are you with the variety of products offered?
    a. Very satisfied
    b. Somewhat satisfied
    c. Neither satisfied or dissatisfied
    d. Somewhat dissatisfied
    e. Very dissatisfied
    f. N/A
11. Are there other sexual health products you would like to see added? (Open-ended)
12. How important do you think it is for Tufts to provide sexual health resources in this way?
    a. Very important
    b. Somewhat important
    c. Neither important or unimportant
    d. Somewhat unimportant
    e. Very unimportant
    f. N/A
13. Is there anything else you’d like to tell us about your experiences with the sexual health vending machine? (Open-ended)

**Part 4: Demographics**

14. What year in school are you?
    a. Freshman (1st year)
    b. Sophomore (2nd year)
    c. Junior (3rd year)
    d. Senior (4th year)
    e. Graduate student
    f. Other: [Open text]
    g. N/A
15. What is your gender identity?
    a. Male
    b. Female
    c. Non-binary / third gender
    d. Prefer to self-describe: [Open text]
    e. N/A
16. What is your sexual identity/orientation?
    a. Straight
    b. Gay
    c. Lesbian
    d. Bisexual
    e. Pansexual
    f. Asexual
    g. Prefer to self describe: [Open text]
    h. N/A
17. What is your race/ethnicity? (Select all that apply)
    a. Asian
    b. Black or African American
    c. Hispanic or Latino
    d. Native American or Alaska Native
    e. Native Hawaiian or Pacific Islander
    f. White
    g. Other: [Open text]
    h. N/A
18. What is your current living situation?
    a. On-campus dorm/apartment
    b. Off-campus - Medford/Somerville area
    c. Off-campus - Commuter
    d. Other: [Open text]
    e. N/A
