# Supplementary material for: Evaluating student use, access, and perceptions of a campus sexual health vending machine
Source: Front Public Health. 2026 Sep 10;14:1916539. doi: 10.3389/fpubh.2026.1916539 (PMC13601293; doi:10.3389/fpubh.2026.1916539)
Supplement: Supplementary file 2 [file Table_1.docx]

**Supplementary Table 1. Summary of main comparative analyses by recent vending machine use.** N varies by item due to exclusion of N/A responses.

| **Outcome** | **Recent users n/N (%)** | **Non-users n/N (%)** | **Fisher’s exact test, p-value** |
| --- | --- | --- | --- |
| Agreement that the vending machine increased likelihood of condom use |  |  |  |
| Agree | 31/37 (83.8%) | 38/64 (59.4%) |  |
| Neither agree nor disagree | 6/37 (16.2%) | 21/64 (32.8%) |  |
| Disagree | - | 5/64 (7.8%) | 0.026 |
| Agreement that the vending machine makes it easier to obtain sexual health products |  |  |  |
| Agree | 39/39 (100%) | 74/76 (97.4%) |  |
| Neither agree nor disagree | - | 2/76 (2.6%) |  |
| Disagree | - | - | 0.548 |
| Satisfaction with the variety of products offered |  |  |  |
| Satisfied | 36/39 (92.3%) | 57/65 (87.7%) |  |
| Neither satisfied or dissatisfied | 2/39 (5.2%) | 8/65 (12.3%) |  |
| Dissatisfied | 1/39 (2.6%) | - | 0.227 |
| Living situation |  |  |  |
| On campus | 27/38 (71.1%) | 45/77 (58.4%) |  |
| Off campus - surrounding area | 10/38 (26.3%) | 31/77 (40.3%) |  |
| Off campus - commuter | 1/38 (2.6%) | 1/77 (1.3%) | 0.268 |

**Supplementary Table 2. Summary of vending machine product satisfaction by demographic characteristic.** N varies by item due to exclusion of N/A responses.

| **Demographic characteristic** | **Satisfied n/N (%)** | **Neither satisfied nor dissatisfied n/N (%)** | **Dissatisfied n/N (%)** | **Fisher’s exact test, p-value** |
| --- | --- | --- | --- | --- |
| Year in school |  |  |  |  |
| Freshman (1st year) | 14/92 (15.2%) | 1/10 (10.0%) | - |  |
| Sophomore (2nd year) | 40/92 (43.5%) | 3/10 (30.0%) | - |  |
| Junior (3rd year) | 18/92 (19.6%) | 5/10 (50.0%) | 1/1 (100.0%) |  |
| Senior (4th year) | 14/92 (15.2%) | 1/10 (10.0%) | - |  |
| Graduate student | 6/92 (6.5%) | - | - | 0.398 |
| Gender identity |  |  |  |  |
| Male | 16/92 (17.45) | 1/9 (11.1%) | - |  |
| Female | 72/92 (78.3%) | 7/9 (77.8%) | 1/1 (100.0%) |  |
| Non-binary | 4/92 (4.3%) | 1/9 (11.1%) | - | 0.685 |
| Sexual identity / orientation |  |  |  |  |
| Heterosexual | 48/87 (55.2%) | 5/9 (55.6%) | 1/1 (100.0%) |  |
| Bisexual / Pansexual | 30/87 (34.5%) | 2/9 (22.2%) | - |  |
| Gay / Lesbian | 8/87 (9.2%) | 2/9 (22.2%) | - |  |
| Other | 1/87 (1.1%) | - | - | 0.594 |
| Race/ethnicity |  |  |  |  |
| White | 48/89 (53.9%) | 4/10 (40.0%) | 1/1 (100.0%) |  |
| Asian | 19/89 (21.3%) | 2/10 (20.0%) | - |  |
| Hispanic / Latino | 11/89 (12.4%) | 2/10 (20.0%) | - |  |
| Black / African American | 2/89 (2.2%) | - | - |  |
| Native Hawaiian / Pacific Islander | 1/89 (1.1%) | - | - |  |
| Other | 8/89 (9.0%) | 2/10 (20.0%) | - | 0.792 |
| Place of residence |  |  |  |  |
| On campus | 59/91 (64.8%) | 5/10 (50.0%) | 1/1 (100.0%) |  |
| Off campus - Surrounding area | 30/91 (33.0%) | 5/10 (50.0%) | - |  |
| Off campus - Commuter | 2/91 (2.2%) | - | - | 0.648 |
